# The role of retinal vessel geometry as an indicator of systemic arterial stiffness assessed by cardio-ankle vascular index

**DOI:** 10.3389/fcvm.2023.1139557

**Published:** 2023-06-19

**Authors:** Dae Joong Ma, Heesun Lee, Ji Min Choi, Hyo Eun Park, Su-Yeon Choi, Hyuk Jin Choi

**Affiliations:** ^1^ Department of Ophthalmology, Hallym University Kangnam Sacred Heart Hospital, Seoul, Republic of Korea; ^2^ Department of Internal Medicine, Seoul National University College of Medicine, Seoul, Republic of Korea; ^3^ Division of Cardiology, Seoul National University Hospital Healthcare System Gangnam Center, Seoul, Republic of Korea; ^4^ Division of Gastroenterology, Seoul National University Hospital Healthcare System Gangnam Center, Seoul, Republic of Korea; ^5^ Department of Ophthalmology, Seoul National University College of Medicine, Seoul, Republic of Korea; ^6^ Department of Ophthalmology, Seoul National University Hospital Healthcare System Gangnam Center, Seoul, Republic of Korea

**Keywords:** vascular stiffness, cardiovascular diseases, cardio-ankle vascular index, retina, vessel

## Abstract

**Objective:**

To determine whether retinal vessel geometry is associated with systemic arterial stiffness, as determined by the cardio-ankle vascular index (CAVI).

**Methods:**

This single-center retrospective cross-sectional study included 407 eyes of 407 subjects who underwent routine health exams, including CAVI and fundus photography. Retinal vessel geometry was measured using a computer-assisted program (Singapore “I” Vessel Assessment). Subjects were classified into two groups based on CAVI values: high CAVI (≥9) or low CAVI (<9). The main outcome measures included the association of retinal vessel geometry and CAVI value evaluated using multivariable logistic regression models.

**Results:**

Three hundred forty-three subjects (343, 84.3%) were in the *low CAVI* group, and 64 (15.7%) subjects were in the high CAVI group. Multivariable logistic linear regression analyses adjusted for age, sex, body mass index, smoking status, mean arterial pressure, and the presence of hypertension, diabetes mellitus, and dyslipidemia showed a significant association between high CAVI values and the following retinal vessel geometry parameters: central retinal arteriolar equivalent caliber (CRAE; adjusted odds ratio [AOR], 0.95; 95% confidence interval [CI], 0.89–1.00*; P* = 0.043), fractal dimension of arteriolar network (FDa; AOR, 4.21 × 10^−4^; 95% CI, 2.32 × 10^−7^−0.77; *P* = 0.042), and arteriolar branching angle (BAa; AOR, 0.96; 95% CI, 0.93–0.99; *P* = 0.007).

**Conclusions:**

Increased systemic arterial stiffness had a significant association with retinal vessel geometry related to arterial narrowing (CRAE), less branching complexity of the arterial tree (FDa), and acute arteriolar bifurcation (BAa).

## 1. Introduction

The retinal vessels are the only part of human circulation that can be directly and noninvasively visualized *in vivo*. Several studies have shown that classic retinal microvascular signs such as microaneurysms, retinal hemorrhages, generalized or focal arteriolar narrowing, and arteriovenous nicking could provide information with respect to the risk of cardiovascular or cerebrovascular events (1). In addition, retinal vessel geometry parameters such as caliber, fractal dimension, tortuosity, and bifurcation angle were quantified using computer-assisted programs and showed associations with cardiovascular or cerebrovascular diseases (2, 3).

Large artery stiffness, which occurs with aging and various pathologic states, delivers excessive pulsatile energy into the microvasculature of target organs that then undergo corresponding damage (4). On the other hand, reduced lumen diameter and rarefaction of small arteries that occur with accelerated aging such essential hypertension, metabolic syndrome, and diabetes, increase total peripheral resistance and mean blood pressure (BP), resulting in increased large-artery stiffness (5). It is proposed that “cross-talk” occurs between large and small arteries, which suggests that small- and large-artery alterations are closely interdependent (6). A growing body of literature addresses the association between large artery stiffness and retinal vasculature as evidence of the cross-talk between large and small arteries (7–13), but all of these studies used pulse wave velocity (PWV) to evaluate large artery stiffness. As BP can affect both PWV and retinal vessel geometry (14–17), the association between retinal vessel geometry and large artery stiffness evaluated using PWV can be overestimated.

To reduce the confounding effect of BP from the cross-talk between large and small arteries, a method independent from BP should be used to evaluate large artery stiffness, such as the cardio-ankle vascular index (CAVI) (18). We therefore aimed to determine whether small retinal vessel geometry is associated with larger systemic arterial stiffness, as determined by CAVI.

## 2. Materials and methods

This single-center retrospective cross-sectional study was conducted at Seoul National University Hospital (SNUH) Healthcare System Gangnam Center in Seoul, Republic of Korea. The institutional review board of SNUH approved this retrospective study (IRB No. H-1907-065-1047), which was conducted in accordance with the Declaration of Helsinki. The IRB waived the requirement for informed consent.

### 2.1. Study population

Four hundred seven subjects who underwent routine health exams, including fundus photography and CAVI, at SNUH Healthcare System Gangnam Center between January 2020 and December 2020 were consecutively enrolled. Patient records and information were anonymized and deidentified prior to analysis. All subjects were 18 years of age or older. The exclusion criteria for the study were as follows: (1) presence of ocular posterior segment disease, which could affect retinal configuration, including age-related macular degeneration, diabetic retinopathy, epiretinal membrane, uveitis, or glaucoma, or (2) eyes with a history of laser treatment or intraocular surgery other than uncomplicated cataract surgery.

### 2.2. Clinical, anthropometric and laboratory measurements

Assessment of current medications, medical history, sociodemographic characteristics, and lifestyle factors, including smoking status, were conducted using a structured self-report questionnaire (19). Smoking status was classified as ever (ex-smoker or current smoker) or never. Body mass index (BMI) was calculated as body weight in kilograms divided by the square of the body height in meters. Obesity was defined as BMI ≥ 25 kg/m^2^. Mean arterial pressure (MAP) was calculated by dividing the sum of the systolic and double diastolic BP by three (20).

Hypertension was defined as (1) systolic BP ≥ 140 mmHg and/or diastolic BP ≥ 90 mmHg, (2) use of antihypertensive medication, or (3) history of hypertension. Diabetes mellitus (DM) was defined as (1) fasting blood sugar level ≥126 mg/dl and/or hemoglobin A1c ≥ 6.5%, (2) use of antidiabetic medications, or (3) history of DM. Dyslipidemia was defined as (1) total cholesterol level ≥240 mg/dl, triglyceride level ≥200 mg/dl, low-density lipoprotein cholesterol ≥160 mg/dl, or high-density lipoprotein <40 mg/dl, (2) use of anti-dyslipidemic medications, or (3) history of dyslipidemia.

### 2.3. Measurement of CAVI

The CAVI was calculated using a VaSera VS-1000 (Fukuda Denshi Co. Ltd, Tokyo, Japan) from the brachial-ankle pulse wave velocity (baPWV) and BP while monitoring the electrocardiogram and heart sounds (21–23). The details of the CAVI and its measurement were previously described (24). In short, with the subject lying supine, cuffs were applied to both upper arms and ankles, electrocardiographic electrodes were attached to both wrists, and a phonocardiogram was placed at the right sternal border in the second intercostal space. The exam was performed after resting for 10 min.

baPWV was calculated by dividing the distance from the aortic valve to the ankle by the summed total of the time between the aortic valve closing sound and the brachial pulse wave notch and between the brachial pulse wave rise and the ankle pulse wave rise.

The CAVI value was obtained by using the following equation:

$$CAVI=a\left[\left(\frac{2\rho }{\Delta P}\right)\times \;ln\left(\frac{Ps}{Pd}\right)\times PWV^{2}\right]+b$$

where *Ps* is systolic BP, *Pd* is diastolic BP, Δ*P* is *Ps*-*Pd*, *ρ* is blood density, and *a* and *b* are constants.

Measurements on the right and left sides of the body (right CAVI and left CAVI) were obtained, and the mean values of the right and left CAVI were calculated for statistical analysis. Using the Japan Society for Vascular Failure criteria, subjects were classified into two groups: “high CAVI”, wherein the CAVI values were abnormal (≥9), and “low CAVI”, wherein the CAVI values were borderline or normal (<9) (25, 26).

### 2.4. Fundus photography and retinal vessel geometry measurements

**Figure 1:**
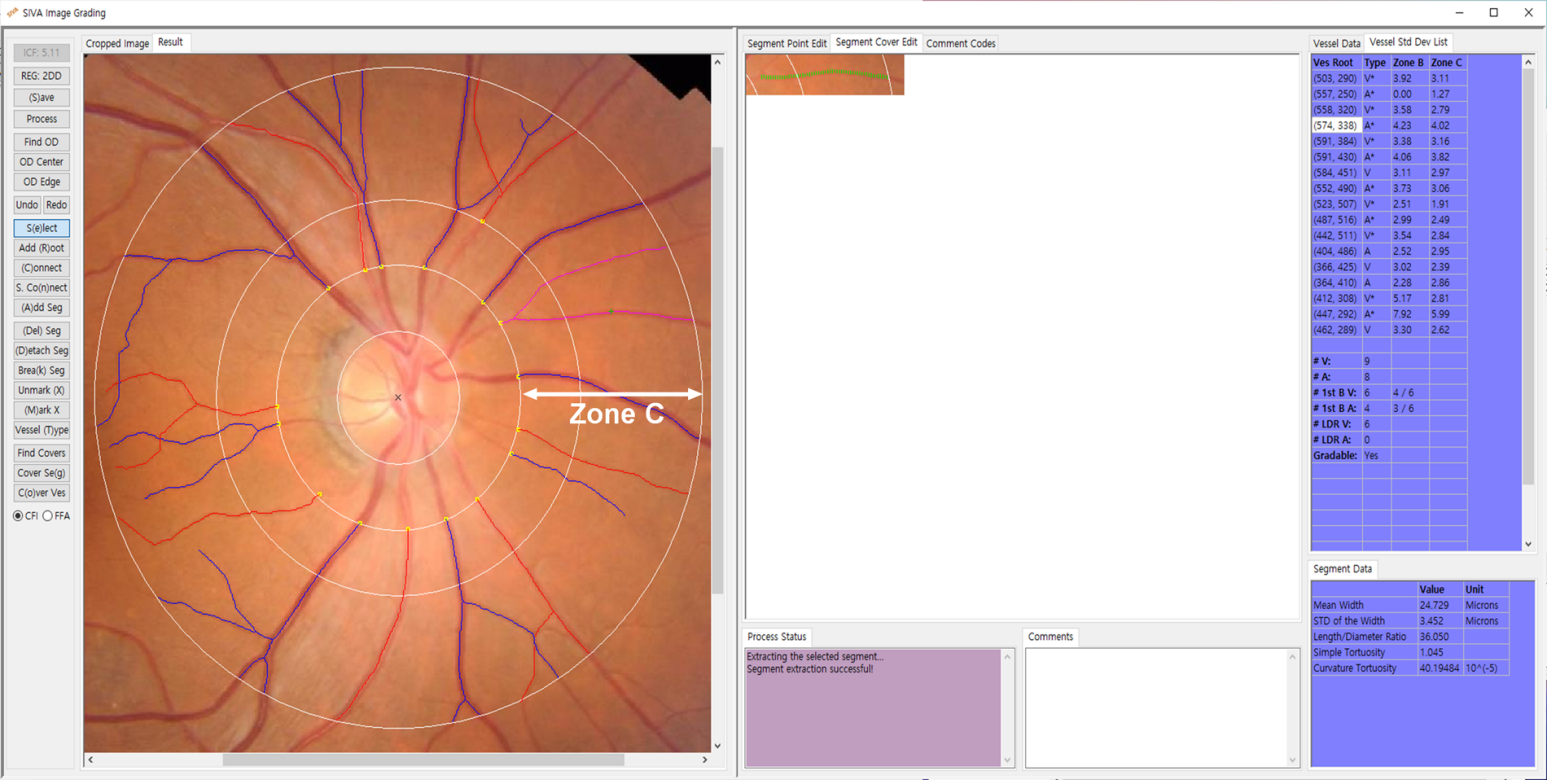
Singapore “I” Vessel Assessment (SIVA) screenshots. The SIVA automatically traces and marks arterioles as red and venules as blue. The arrow indicates a circular region between the 2nd and 5th optic disc radii from the center of the optic disc in which the measurements are made (Zone C).

Digital fundus photographs were acquired from participants using a 45° non-mydriatic retinal camera (TRC-NW8, Topcon Inc., Tokyo, Japan). Retinal vessel geometry was measured using semiautomated software [Singapore “I” Vessel Assessment (SIVA)—cloud-based version, National University of Singapore, Singapore] by two graders (H.J.C. and D.J.M.) following the developer's protocol (27). As substantial correlations of the retinal vessel geometry between the right and left eyes have been reported (28), the best quality image (either right or left) for each participant was analyzed. SIVA automatically identified and traced arterioles and venules. Then, the grader manually corrected vessel tracing and removed artifacts. SIVA automatically generated retinal vessel geometry parameters. The circular region between the 2nd and 5th radii of the optic disc from the center of the optic disc (Zone C) was evaluated (Figure 1). The retinal vessel geometry parameters analyzed in the present study are listed in Table 1. The average of the parameters evaluated by the two graders was used in further analysis.

**Table 1:** Description of the 15 retinal vessel geometry parameters analyzed using the Singapore “I” Vessel Assessment (SIVA) (34).

Parameter	Description	
CRAE	Central retinal arteriolar equivalent caliber	Central retinal artery/vein equivalent width determined using the revised Knudtson-Parr–Hubbard formula [29].
CVAE	Central retinal venular equivalent caliber	
AVR	Arteriole–venule ratio	The ratio of CRAE to CRVE.
FDa	Fractal dimension of arteriolar network	Summary measure of the branching complexity of the retinal vascular tree.
FDv	Fractal dimension of venular network	
MWa	Mean width of arteriole	Average width of the sample vessel segments.
MWv	Mean width of venule	
STDWa	Standard deviation of arteriole width	Standard deviation of the width from the sample vessel segments.
STDWv	Standard deviation of venule width	
sTORTa	Arteriolar simple tortuosity	Arc-chord ratio of the sample vessel segments.
sTORTv	Venular simple tortuosity	
cTORTa	Arteriolar curvature tortuosity	Integral of the curvature squared along the path of the sample vessel segment, normalized by the total length of the path.
cTORTv	Venular curvature tortuosity	
BCa	Arteriolar branching coefficient	Summed square of the mean width of the two daughter vessels, divided by the square of the mean width of the parent vessel.
BCv	Venular branching coefficient	
JEa	Junctional exponent deviation for arterioles	Deviation of the junctional exponent from the Murray's law prediction which reflects the optimum ratio of vessel widths at a bifurcation.
JEv	Junctional exponent deviation for venules	
BAa	Arteriolar branching angle	The angle subtended between two daughter vessels at a vascular bifurcation.
BAv	Venular branching angle	
AAa	Arteriolar angular asymmetry	The difference in the two daughter angles of the first-order branch.
AAv	Venular angular asymmetry	
LDRa	Arteriolar length-to-diameter ratio	The ratio of the segment length between the branches to its average diameter.
LDRv	Venular length-to-diameter ratio	

### 2.5. Statistical analysis

Data are expressed as the mean [standard deviation (SD)] [range] or numbers (percentages). Student's *t* test was used for the comparison of the continuous variables between the groups. A univariable analysis was performed to evaluate the associations between CAVI values and retinal vessel geometry parameters and conventional cardiovascular risk factors. Then, we assessed whether CAVI values were associated with retinal vessel geometry parameters using multivariable logistic regression analyses after adjusting for conventional cardiovascular risk factors. All variables with *P* < 0.10 in the univariable analysis were included in a multivariable logistic regression model. Adjusted odds ratios (AORs) and 95% confidence intervals (CIs) were used to assess the strength of the association.

The level of statistical significance was set as *P *< 0.05. Data were analyzed using SPSS 22.0 software for Windows (SPSS Inc., Chicago, IL).

## 3. Results

### 3.1. Clinical, anthropometric and laboratory characteristics

A total of 407 eyes of 407 subjects were analyzed. The characteristics of the study population are shown in Table 2. The mean (SD) BMI was 24.1 (3.1) kg/m^2^, and 36.4% of the total population was obese. Approximately half of the subjects (58.0%) were current or previous smokers. In terms of comorbidities, 36.4%, 12.8%, and 31.0% of the total population had hypertension, DM, and dyslipidemia, respectively; these rates were similar to the prevalence in Korean adults (30–32).

**Table 2:** Clinical characteristics of the study population.

Characteristics	Total	CAVI < 9	CAVI ≥ 9	*P* value^a^
	(*n* = 407)	(*n* = 343)	(*n* = 64)	
Cardio-ankle vascular index, mean (SD) [range]	7.94 (1.0)	7.6 (0.7)	9.7 (0.6)	<0.001
	[5.7–11.8]	[5.7–9.0]	[9.0–11.8]	
Clinical data				
Age, years, mean (SD) [range]	57.1 (8.9)	55.5 (8.2)	65.6 (7.47)	<0.001
	[30.2–80.2]	[30.2–77.1]	[45.8–80.2]	
Sex, male, *n* (%)	292 (71.7%)	243 (70.8%)	49 (76.6%)	0.351
Hypertension, *n* (%)	148 (36.4%)	116 (33.8%)	32 (50.0%)	0.013
Diabetes mellitus, *n* (%)	52 (12.8%)	34 (9.9%)	18 (28.1%)	<0.001
Dyslipidemia, n	126 (31.0%)	97 (28.3%)	29 (45.3%)	0.007
Obesity, *n* (%)	148 (36.4%)	127 (37.0%)	21 (32.8%)	0.520
Smoking status, *n* (%)				
Never smoker	171 (42.0%)	149 (43.4%)	22 (34.2%)	0.017
Ex- or current smoker	236 (58.0%)	194 (56.6%)	42 (65.6%)	
Anthropometric data				
Mean arterial pressure, mmHg, mean (SD) [range]	100.5 (10.9)	99.6 (10.6)	105.4 (11.0)	<0.001
	[72.7–146.0]	[72.7–141.7]	[80.0–146.0]	
Body mass index, kg/m^2^, mean (SD) [range]	24.1 (3.1)	24.1 (3.1)	24.0 (2.8)	0.899
	[16.4–34.2]	[16.4–34.2]	[16.4–29.9]	
Laboratory data				
Fasting glucose, mg/dl, mean (SD) [range]	105.5 (20.8) [51.0–235.0]	103.6 (19.5) [72.0–235.0]	115.6 (24.5) [51.0–195.0]	0.002
Total cholesterol, mg/dl, mean (SD) [range]	189.4 (39.4) [88.0–298.0]	190.7 (39.8) [88.0–298.0]	182.3 (36.5) [97.0–256.0]	0.117
Triglyceride, mg/dl, mean (SD) [range]	124.4 (82.5) [27.0–537.0]	124.0 (83.3) [27.0–537.0]	126.9 (78.8) [50.0–500.0]	0.792
HDL-cholesterol, mg/dl, mean (SD) [range]	52.3 (12.6) [29.0–119.0]	52.8 (12.8) [29.0–119.0]	49.6 (11.1) [30.0–86.0]	0.061
LDL-cholesterol, mg/dl, mean (SD) [range]	120.9 (35.6) [32.0–241.0]	121.7 (35.3) [32.0–241.0]	116.9 (36.9) [34.0–186.0]	0.330
HbA1c, %, mean (SD) [range]	5.9 (0.7) [4.7–10.5]	5.8 (0.7) [4.70–10.50]	6.1 (0.6) [5.1–8.5]	0.002
BUN, mg/dl, mean (SD) [range]	14.1 (3.6) [7.0–27.0]	13.7 (3.5) [7.0–27.0]	15.9 (4.0) [7.0–25.0]	<0.001
Creatinine, mg/dl, mean (SD) [range]	0.88 (0.19) [0.48–2.00]	0.87 (0.18) [0.48–2.00]	0.93 (0.23) [0.54–1.45]	0.002

^a^ CAVI < 9 vs. CAVI ≥ 9.

BUN, blood urea nitrogen; CAVI, cardio-ankle vascular index; LDL, cholesterol low-density lipoprotein cholesterol; HbA1c, glycosylated hemoglobin; HDL, cholesterol high-density lipoprotein cholesterol; SD, standard deviation.

Among them, 343 (84.3%) subjects were in the low CAVI group, and 64 (15.7%) subjects were in the high CAVI group (Table 2). The mean (SD) CAVI was 7.6 (0.7) in the low CAVI group and 9.7 (0.6) in the high CAVI group. The prevalence of hypertension, DM, dyslipidemia, and smoking history (ex- or current smoker) was significantly higher in the high CAVI group than in the low CAVI group (*P *= 0.013, < 0.001, 0.07, and 0.017, respectively). MAP was also significantly higher in the high CAVI group than in the low CAVI group (*P *< 0.001). Significant differences were not observed in sex composition, body mass index, or the prevalence of obesity between the two groups.

Values for fasting glucose, glycosylated hemoglobin (HbA1c), blood urea nitrogen, and creatinine were significantly higher in the high CAVI group. There were no significant differences in total cholesterol, triglycerides, high-density lipoprotein cholesterol, or low-density lipoprotein cholesterol between the two groups (Table 2).

### 3.2. Association of CAVI with conventional risk factors

For the univariable logistic regression analysis, age [odds ratio (OR), 1.17; 95% CI, 1.12–1.22; *P *< 0.001], presence of hypertension (OR, 1.96; 95% CI, 1.14–3.35; *P* = 0.015), DM (OR, 3.56; 95% CI, 1.86–6.81; *P *< 0.001), dyslipidemia (OR, 2.10; 95% CI, 1.22–3.63; *P *= 0.008), MAP (OR, 1.05; 95% CI, 1.02–1.08; *P *< 0.001), and HbA1c (OR, 1.68; 95% CI, 1.19–2.37; *P* = 0.003) were found to be significantly associated with high CAVI values (Table 3). Sex, BMI, obesity, and smoking history were not significantly associated with high CAVI values.

**Table 3:** Univariable analysis of conventional risk factors with respect to high cardio-ankle vascular index (CAVI) level (CAVI ≥ 9).

Risk factors	OR (95% CI)	*P* value
Age, years	1.17 (1.12–1.22)	<0.001
Sex, male	1.34 (0.72–2.51)	0.352
Hypertension	1.96 (1.14–3.35)	0.015
Diabetes mellitus	3.56 (1.86–6.81)	<0.001
Dyslipidemia	2.10 (1.22–3.63)	0.008
Mean arterial pressure	1.05 (1.02–1.08)	<0.001
Body mass index	0.99 (0.91–1.08)	0.898
Obesity	1.20 (0.68–2.12)	0.520
Ex- or current smoker	0.68 (0.39–1.19)	0.179
HbA1c, %,	1.96 (1.14–3.35)	0.015

CI, confidence interval; HbA1c, glycosylated hemoglobin; OR, odds ratio.

### 3.3. Association of CAVI with retinal vessel geometry parameters

The retinal vessel geometry parameters with respect to the CAVI levels in eligible subjects are shown in Supplementary Table S1.

Univariable logistic linear regression analysis revealed that a high CAVI value was signiﬁcantly associated with central retinal arteriolar equivalent caliber (CRAE; OR, 0.96; 95% CI, 0.92–1.00; *P *= 0.055), arteriole–venule ratio (OR, 0.04; 95% CI, 1.21 × 10^−4^−12.96; *P *= 0.040), fractal dimension of arteriolar network (FDa; OR, 1.00 × 10^−6^; 95% CI, 3.10 × 10^−9^−4.83 × 10^−4^; *P *< 0.001), fractal dimension of venular network (OR, 4.46 × 10^−3^; 95% CI, 1.40 × 10^−5^−1.39; *P *= 0.065), arteriolar curvature tortuosity (OR, 0.00; 95% CI, 0.00–4.12 × 10^146^; *P* = 0.057), arteriolar branching angle (BAa; OR, 0.96; 95% CI, 0.93–0.98; *P* < 0.001), arteriolar angular asymmetry (OR, 0.98; 95% CI, 0.96–1.00; *P *= 0.066), and venular angular asymmetry (OR, 0.98; 95% CI, 0.96–1.00; *P *= 0.018) (Table 4).

**Table 4:** Univariable and multivariable analysis of retinal vessel geometry evaluated with Singapore “I” Vessel Assessment associated with high cardio-ankle vascular index (CAVI) levels (CAVI ≥ 9).

	Univariable		Multivariable^a^	
	OR (95% CI)	*P* value	AOR (95% CI)	*P* value
CRAE	0.96 (0.92–1.00)	0.055	0.95 (0.89–1.00)	0.043
CVAE	0.99 (0.96–1.02)	0.461		
AVR	0.04 (1.21 × 10^−4^−12.96)	0.040	0.02 (2.40 × 10^−5^−24.83)	0.293
FDa	1.00 × 10^−6^ (3.10 × 10^−9^−4.83 × 10^−4^)	<0.001	4.21 × 10^−4^ (2.32 × 10^−7^−0.77)	0.042
FDv	4.46 × 10^−3^ (1.40 × 10^−5^−1.39)	0.065	0.05 (2.30 × 10^−5^−73.47)	0.403
MWa	0.99 (0.94–1.05)	0.800		
MWv	1.01 (0.96–1.06)	0.681		
STDWa	1.04 (0.92–1.16)	0.553		
STDWv	1.06 (0.92–1.23)	0.415		
sTORTa	1.3 × 10^−7^ (3.73 × 10^−16^−46.14)	0.114		
sTORTv	688.45 (0.02–1.84 × 10^7^)	0.212		
cTORTa	0.00 (0.00–4.12 × 10^146^)	0.057	0.00 (0.00)	0.509
cTORTv	0.00 (0.00)	0.609		
BCa	0.96 (0.43–2.15)	0.922		
BCv	0.90 (0.31–2.57)	0.838		
JEa	1.06 (0.51–2.24)	0.872		
JEv	1.00 (0.45–2.21)	0.990		
BAa	0.96 (0.93–0.98)	<0.001	0.96 (0.93–0.99)	0.007
BAv	0.98 (0.96–1.01)	0.142		
AAa	0.98 (0.96–1.00)	0.066	0.99 (0.96–1.02)	0.498
AAv	0.98 (0.96–1.00)	0.018	0.99 (0.96–1.01)	0.251
LDRa	1.00 (0.95–1.05)	0.917		
LDRv	0.99 (0.94–1.04)	0.581		

^a^ Adjusted for age, sex, body mass index, smoking status, mean arterial pressure, and the presence of obesity, hypertension, diabetes mellitus and dyslipidemia.

AAa, Arteriolar angular asymmetry; AAv, Venular angular asymmetry; AOR, Adjusted odds ratio; AVR, Arteriole–venule ratio; BAa, Arteriolar branching angle; BAv, Venular branching angle; BCa, Arteriolar branching coefficient; BCv, Venular branching coefficient; CI, Confidence interval; CRAE, Central retinal arteriolar equivalent caliber; cTORTa, Arteriolar curvature tortuosity; cTORTv, Venular curvature tortuosity; CVAE, Central retinal venular equivalent caliber; FDa, Fractal dimension of arteriolar network; FDv, Fractal dimension of venular network; JEa, Junctional exponent deviation for arterioles; JEv, Junctional exponent deviation for venules; LDRa, Arteriolar length-to-diameter ratio; LDRv, Venular length-to-diameter ratio; MWa, Mean width of arteriole; MWv, Mean width of venule; OR, Odds ratio; STDWa, Standard deviation of arteriole width; STDWv, Standard deviation of venule width; sTORTa, Arteriolar simple tortuosity; sTORTv, Venular simple tortuosity.

However, in the multivariable logistic linear regression analysis after adjusting for conventional risk factors, including age, sex, BMI, MAP, smoking history, and the presence of obesity, hypertension, DM and dyslipidemia, high CAVI values were signiﬁcantly associated with decreased CRAE [adjusted OR (AOR), 0.95; 95% CI, 0.89–1.00; *P* = 0.043], FDa (AOR, 4.21 × 10^−4^; 95% CI, 2.32 × 10^−7^−0.77; *P* = 0.042), and BAa (AOR, 0.96; 95% CI, 0.93–0.99; *P *= 0.007), which maintained independent associations with high CAVI values. This indicates that a high CAVI value was associated with narrower retinal arterioles (CRAE), less complex branching pattern (FDa), and narrower bifurcation angle (BAa).

## 4. Discussion

In this retrospective cross-sectional study of 407 routine health examinees, we evaluated the association between retinal vessel geometry and systemic arterial stiffness assessed by CAVI. CRAE, FDa, and BAa were significantly associated with systemic arterial stiffness, even after adjusting for conventional cardiovascular risk factors.

Through cross-talk between the macro- and the microcirculation, large artery stiffness is associated with retinal vasculature. A large body of literature has reported the association between large artery stiffness and retinal vessel caliber, but the results are inconsistent (7–12). In contrast, only a few studies evaluated retinal vessel geometry other than vascular caliber (12, 13). However, all of these studies used PWV to evaluate large artery stiffness, which is known to depend on BP at the time of measurement (14).

As a result of the measurement mechanism of PVW, an increase in blood pressure (BP) at the time of measurement can cause a corresponding increase in the PVW value within the same patient, and conversely, a decrease in BP can lead to a decrease in PVW value (14). As large artery stiffness is also positively correlate with BP, the measurement of large artery stiffness using PVW may overestimate the effect of BP. Likewise, BP is negatively associated with retinal arteriolar diameter (15), retinal vascular fractal dimension (16), and retinal arteriolar branching angle (17). This suggests that the effect of BP can overestimate the association between retinal vessel geometry and large artery stiffness measured by PWV. For large artery stiffness measured using PWV, the association between large artery stiffness and retinal vessel geometry may originate from the confounding effect of BP.

To eliminate possible confounding effects of BP, it is necessary to measure large artery stiffness in a way that is independent of BP, such as CAVI. CAVI is a novel systemic arterial stiffness index that offers better reproducibility than PWV (24). Furthermore, unlike PWV, CAVI is independent of BP. To the best of our knowledge, this is the first study to investigate the association between CAVI and various retinal vascular geometries. Using CAVI, we can eliminate the effect of BP from the association between systemic arterial stiffness and retinal vessel geometry.

In the present study, a high CAVI value showed a significant negative association with CRAE, which indicated the narrowing of retinal arterioles. This finding was consistent with a previous study that showed a negative association between PWV and CRAE (9). This is evidence of the cross-talk between the large artery and the retinal arteries. Increased arterial pulsatility due to large-artery stiffening can be transmitted into retinal arteries. This is followed by remodeling with progressive encroachment of the retinal arterial lumen, aimed at protecting against pulsatile stress. Reduced lumen diameter of the small resistance arteries is a major determinant of the increase in total peripheral resistance and MAP, which in turn increases large-artery stiffness.

We additionally demonstrate that a high CAVI value is associated with decreased FDa, indicating less branching complexity, means rarefaction. This is in accordance with previous studies that showed a negative association between PWV and retinal vascular fractal dimension (13), However, these previous studies did not distinguish arterial and venular fractal dimensions. In this study, we could distinguish that CAVI is associated with arteriolar fractal dimension rather than venular fractal dimension. This is additional evidence of the cross-talk between the large artery and the retinal arteries that highlights elevated central systolic BP and pulse pressure (PP) due to the increased large-artery stiffness resulting in the rarefaction of the small retinal arteries. This is followed by increased total peripheral resistance and MAP that results in increases in large-artery stiffness (5).

Furthermore, a high CAVI value was found to have a significant negative association with BAa, which indicates narrower retinal vascular bifurcation angles. Although the relationship between large artery stiffness and vascular bifurcation angle stress is not entirely clear, the following potential explanations may be considered from the viewpoint of the cross-talk between the large artery and coronary artery. The bifurcation angle of the coronary artery strongly alters its mechanical stress distribution under PP. High tensile and low oscillatory shear stress simultaneously occur at the wider-angled bifurcation (33). As higher PP due to the increased large artery stiffness may result in the high tensile stress, which is hypothesized to cause stenosis, the bifurcation angle is narrowed to avoid stenosis in coronary arteries.

The rationale of our study is based on a widely accepted concept that the evaluation of the various retinal vessel geometries can provide further information on the microvascular changes in the heart, brain, and kidney. The exact mechanism of these associations was not elucidated, but some researchers suggest that retinal arterioles share anatomical and physiological characteristics with cardiac, cerebral, and renal arterioles (34). However, cross-talk between large arteries and small arteries may be a more plausible explanation, which is partly evidenced by the present study. As fundus photography is inexpensive, noninvasive, rapid, and widely available, the use of retinal vessel geometry as a mass screening tool for large artery stiffness can be considered.

The present study included some limitations. First, due to its retrospective and cross-sectional design, this study does not show the temporality and causality in the observed associations. However, small- and large-artery alterations are closely interdependent in both physiological and pathological conditions, which makes a temporal relationship difficult to establish (6). Therefore, the importance of temporality and causality in the evaluation of the association of retinal vessel geometry and large artery stiffness is limited. In addition, we could not include the refractive error and axial length in the analysis, which may influence the retinal vessel geometry. Finally, there may be residual uncontrolled confounding factors that may bias or modify the associations observed in our study. Therefore, a prospective and longitudinal study is required to confirm the pathophysiological mechanisms linking large artery stiffness and retinal vascular geometric changes after adjusting for further possible confounders.

In conclusion, this study identifies an association between retinal vessel geometry and systemic arterial stiffness determined by CAVI that is independent of BP, which may provide high-quality evidence of cross-talk between small and large arteries. Our findings provide evidence for the analysis of retinal vessel geometry from fundus photography can be used as an indicator of large artery stiffness. Further prospective studies are required to confirm whether the geometry of retinal vessels can serve as a predictive tool for cardiovascular events and mortality.

## Data Availability

The datasets used and analyzed during the current study are available from the corresponding author on reasonable request.
