# Supplementary material for: The role of retinal vessel geometry as an indicator of systemic arterial stiffness assessed by cardio-ankle vascular index
Source: Front Cardiovasc Med. 2023 Jun 19;10:1139557. doi: 10.3389/fcvm.2023.1139557 (PMC10321710; doi:10.3389/fcvm.2023.1139557)
Supplement: Supplementary file 1 [file Table1.docx]

**Supplementary Material**

**Supplementary Table 1. Baseline characteristics of study subjects according to cardio-ankle vascular index level.**

| **Characteristics** | **CAVI <9**  **(n = 343)** | **CAVI ≥9**  **(n = 64)** | ***P value*** |
| --- | --- | --- | --- |
| **CRAE, mean (SD) [range]** | 118.7 (6.8) [98.4-141.3] | 117.0 (6.3) [98.7-130.1] | 0.054 |
| **CVAE, mean (SD) [range]** | 164.8 (10.3) [137.6-198.6] | 163.8 (8.5) [145.8-191.3] | 0.462 |
| **AVR, mean (SD) [range]** | 0.72 (0.05) [0.57-0.89] | 0.72 (0.04) [0.60-0.82] | 0.275 |
| **FDa, mean (SD) [range]** | 1.19 (0.05) [1.04-1.33] | 1.16 (0.05) [1.04-1.25] | <0.001 |
| **FDv, mean (SD) [range]** | 1.19 (0.05) [1.00-1.34] | 1.18 (0.04) [1.08-1.26] | 0.064 |
| **MWa, mean (SD) [range]** | 62.9 (4.4) [46.5-80.7] | 62.7 (5.1) [48.0-73.3] | 0.801 |
| **MWv, mean (SD) [range]** | 69.9 (5.5) [58.6-88.8] | 70.2 (4.4) [61.0-80.6] | 0.682 |
| **STDWa, mean (SD) [range]** | 10.4 (2.3) [5.4-19.9] | 10.5 (2.3) [6.2-15.4] | 0.554 |
| **STDWv, mean (SD) [range]** | 8.8 (1.8) [4.9-14.7] | 9.0 (1.6) [5.3-12.6] | 0.416 |
| **sTORTa, mean (SD) [range]** | 1.09 (0.02) [1.06-1.16] | 1.08 (0.01) [1.06-1.16] | 0.114 |
| **sTORTv, mean (SD) [range]** | 1.09 (0.02) [1.06-1.26] | 1.10 (0.04) [1.07-1.33] | 0.398 |
| **cTORTa, mean (SD) [range]** | 1.4x10^-4^ (2.6x10^-5^) [6.8x10^-5^-2.6x10^-4^] | 1.3x10^-4^ (2.7x10^-5^) [7.8x10^-5^-2.1x10^-4^] | 0.057 |
| **cTORTv, mean (SD) [range]** | 1.4x10^-4^ (2.7x10^-5^) [8.4x10^-5^-2.4x10^-4^] | 1.4x10^-4^ (3.4x10^-5^) [0.0-2.2x10^-4^] | 0.610 |
| **BCa, mean (SD) [range]** | 1.66 (0.33) [0.95-3.72] | 1.66 (0.33) [1.14-3.00] | 0.922 |
| **BCv, mean (SD) [range]** | 1.49 (0.26) [0.44-2.90] | 1.49 (0.21) [1.06-2.28] | 0.838 |
| **JEa, mean (SD) [range]** | -0.57 (0.36) [-1.37-0.69] | -0.57 (0.36) [-1.25-0.50] | 0.872 |
| **JEv, mean (SD) [range]** | -0.45 (0.35) [-1.40-0.93] | -0.45 (0.31) [-1.02-0.43] | 0.990 |
| **BAa, mean (SD) [range]** | 77.7 (11.7) [32.9-112.7] | 71.0 (12.8) [30.0-101.4] | <0.001 |
| **BAv, mean (SD) [range]** | 78.7 (10.5) [40.5-117.1] | 76.5 (13.6) [51.4-133.0] | 0.142 |
| **AAa, mean (SD) [range]** | 37.4 (12.5) [3.0-80.5] | 34.3 (12.7) [3.0-61.1] | 0.065 |
| **AAv, mean (SD) [range]** | 41.9 (13.2) [3.2-78.8] | 37.5 (13.9) [2.1-70.7] | 0.022 |
| **LDRa, mean (SD) [range]** | 12.4 (6.0) [1.2-38.5] | 12.3 (5.5) [2.8-26.2] | 0.917 |
| **LDRv, mean (SD) [range]** | 11.9 (5.9) [0.8-34.3] | 11.4 (6.0) [1.7-27.3] | 0.582 |

*AAa,* Arteriolar angular asymmetry; *AAv* ,Venular angular asymmetry; *AOR,* Adjusted odds ratio; *AVR,* Arteriole–venule ratio; *BAa,* Arteriolar branching angle; *BAv,* Venular branching angle; *BCa,* Arteriolar branching coefficient; *BCv,* Venular branching coefficient; *CI,* Confidence interval; *CRAE,* Central retinal arteriolar equivalent caliber; *cTORTa,* Arteriolar curvature tortuosity; *cTORTv,* Venular curvature tortuosity; *CVAE,* Central retinal venular equivalent caliber; *FDa,* Fractal dimension of arteriolar network; *FDv,* Fractal dimension of venular network; *JEa,* Junctional exponent deviation for arterioles; *JEv,* Junctional exponent deviation for venules; *LDRa,* Arteriolar length-to-diameter ratio; *LDRv,* Venular length-to-diameter ratio; *MWa,* Mean width of arteriole; *MWv,* Mean width of venule; *OR,* Odds ratio; *STDWa,* Standard deviation of arteriole width; *STDWv,* Standard deviation of venule width; *sTORTa,* Arteriolar simple tortuosity; *sTORTv,* Venular simple tortuosity.
